# Effect of lycopene as an adjuvant therapy with 5-florouracil in human colon cancer

**DOI:** 10.1016/j.sjbs.2022.103392

**Published:** 2022-07-25

**Authors:** Norah M. Alhoshani, Norah S. Al-Johani, Nora Alkeraishan, Saud Alarifi, Saad Alkahtani

**Affiliations:** Department of Zoology, College of Science, King Saud University, P.O. Box 2455, Riyadh 11451, Saudi Arabia

**Keywords:** Cancer, Colon, Lycopene, 5-Fluorouracil, Cytotoxicity, Apoptosis

## Abstract

Colon cancer (CC) is among the most frequent human cancers. Although, there is improvement in diagnostic techniques and existing treatment possibilities. Still, there is an unmet need for a novel treatment regimen that will improve the patient's quality of life. Here, the role of lycopene as an adjuvant therapy with 5-fluorouracil (5-FU) was explored in Caco2 colon cancer cells. Cells were exposed to a dose (3 µg/ml) of 5-FU and three doses (60, 90, 120 µg/ml) of lycopene either alone or as a mixture with 5-FU. Cytotoxicity, genotoxicity, oxidative stress, gene expression, and apoptotic parameters were investigated in this study. Findings showed that 5-FU or lycopene alone induced a dose-dependent increase in cytotoxicity which was slightly reduced in lycopene mixtures. Apoptotic assays showed that 5-FU induced a significant level of apoptosis but not necrosis. However, a lycopene mixture with 5-FU enhanced 5-FU triggered apoptosis and promoted necrosis. The mixtures were also shown to suppress mitochondrial membrane potential while gene expression analyses showed the induction of Bax expression upon exposure to mix 90 exhibited the highest Bax to Bcl-2 ratio and caspase 3 and 9 gene expression. Furthermore, the mixture treatment also inhibited cell migration in the wound healing assay compared to 5-FU alone. In conclusion, lycopene was found to sensitize Caco 2 cell lines to 5-FU treatment by inducing the expression of apoptotic genes. This, coupled with lycopene suppression of cytotoxicity and cell migration, indicates lycopene may be a promising candidate for adjuvant therapy involving 5-FU in CC.

## 1 Introduction

Colon cancer (CC) is amongst the most common human cancers, it represents about 10 % of the global cancer incidence and 9.4 % of global cancer mortality as of 2020 (Sung et al., 2021). Recent developments in the knowledge base of CC and research producing a broad array of therapeutic options have improved patients’ overall survival. Unfortunately, there is a continued rise in the number of global new CC cases, which is predicted to increase to 3.2 million individuals by 2040 (Xi and Xu, 2021). Even with the improved diagnosis and available treatment modalities, the rise in the number of new cases will only influence the mortality rates, necessitating the need for better therapeutic strategies that better improve patients’ overall survival to alleviate the rise in the number of deaths. While there is a report of decreased incidence of CC in elderly patients, especially in the 50 to 75-year age group, there is an increased incidence in the younger population between 20 and 39 years, likely as a consequence of enhanced westernization of the diet (Siegel et al., 2019). This is an especially worrisome scenario for Saudi Arabia due to the young population, with about 86 % of the population younger than 50 years (Alyabsi et al., 2021).

Furthermore, risk factors for CC such as obesity, smoking tobacco, and physical inactivity are prevalent in Saudi Arabia (Al-Zalabani, 2020), which can be an indication of increased future incidence of CC and increased financial burden to the government. Conventional treatments for locally advanced CC include surgical resection or excision of tumors, usually followed by adjuvant immunotherapy, chemotherapy, or radiotherapy. However, there are some setbacks to some of these treatment options. Immunotherapy involving the use of autologous tumor cell vaccine is only beneficial to a specific set of patients, while peptide vaccines may induce a negative immune response in some patients (Xiang et al., 2013). Adjuvant chemotherapeutics such as oxaliplatin combined with 5-FU or capecitabine are the standard adjuvant chemotherapy for CC (Lund et al., 2016, Soveri et al., 2019). However, oxaliplatin is known to induce neurotoxicity that causes hyper-excitation of axons resulting in oxidative stress and neuronal damage (Soveri et al., 2019, Duran et al., 2020). In addition, 5-FU also causes unpleasant side effects such as cardiotoxicity, alopecia, bone marrow suppression, which results in neutropenia and inability to fight infections, and gastro-intestinal upsets, including stomatitis, vomiting, nausea, and diarrhoea (Opattova et al., 2019). As such, there is an unmet need for effective adjuvant chemotherapy with little or no adverse effects for patients with CC.

Natural bioactive compounds like flavonoids and terpenes have been explored in recent years for antioxidant activities in different settings. For instance, there are different studies demonstrating anticancer properties of some flavonoids present in Cannabis, such as kaempferol, showed to decrease the proliferation of cancer cells through induction of autophagy (Zhang and Ma, 2019), cannflavin, which induces apoptosis in pancreatic cancer (Moreau et al., 2020) and luteolin, which induces apoptosis breast cancer (Gao et al., 2019). Lycopene is a carotenoid and it has been shown to possess antioxidant characteristics. Lycopene is found in pink grapefruit, watermelons, tomato, and tomato-based food products. There is a variety of research establishing lycopene's anti-cancer effects. For example, one study demonstrated that increasing lycopene consumption lowers the risk of developing certain cancers, such as cervical and ovarian cancer (Ghadage et al., 2019). Consumption of 200 to 400 mg/kg of lycopene was shown to reduce ovarian cancer incidence and tumor size in an *in vivo* model (Sahin et al., 2018). To modulate the effect of 5-FU as an anticancer drug, we test the potential protective effect of lycopene as an adjuvant therapy to improve the 5-FU cancer-killing impact on a Caco2 cell line and suppress the associated adverse effects of 5-FU.

## 2 Material and methods

### 2.1 Cell culture

Caco2 cells were obtained from American Type Culture Collection ATCC, USA, and were cultured in 10 % supplemented medium Dulbecco,s modification of eagles DMEM with Fetal Bovine Serum FBS in addition to 1 % Penicillin at 37 °C in Humidified atmosphere containing 5 % CO_2_. Based on experiments, cells were grown in 96, 24 well plate, 25- T, or 75- T culture flasks. The exponentially growing cells were washed with PBS and harvested by trypsin to spun down at 1600 rpm for five min at 4 °C then cells were diluted with medium at a density of 5 × 10^5^ cells/ml.

### 2.2 MTT assay

Cell viability was evaluated according to Mosmann (1983). Briefly, 5 × 10^5^ cells/ml were seeded, followed by 24 hrs incubation. Afterwards, the cells were exposed to 5-FU (0.5, 1, 2, 4, 8, 16 µg/ml) and Lycopene (70, 100, 125, 175, 200 µg/ml) for 24 hrs. MTT reagent was added for 2 hrs at 37 °C, followed by shaking for 15 min before absorbance measurement at 540 nm in a spectrophotometer (Synergy-H1; BioTek). IC_50_ values were computed using Origin software (Oka et al., 1992).

### 2.3 Natural Red Uptake (NRU) assay

According to Borenfreund and Puerner, (1985), 100 µl of the medium at density 5 × 10^5^ of cells were cultivated and incubated for 24 h. The cells were exposed to 3 µg/ml of 5-FU, 60, 90, and 120 µg/ml of lycopene, and the mixture of the 5-FU plus each dose of lycopene was followed by a 24 hrs incubation. 200 µl per well of NRU dye was added, followed by incubation for 3 hrs at 37 °C. The cells were then fixed with 100 µl of 0.5 g of CaCl2. Destain step was carried out on a shaker for 15 min. OD was measured at 540 nm in a microplate spectrophotometer (Ali et al., 2020).

### 2.4 Wound healing assay

Caco2 cells were seeded in a 12-well plate and incubated at 37 °C for 24 hrs. A long, straight scratch was made in the middle of the well using a pipette tip. To evaluate cell migration ratio, the cells were exposed to 5-FU, 60, 90, and 120 µg/ml of lycopene, and the mixture as before. Cell growth was measured under a microscope connected with a digital MC-170 HD camera (Leica, Germany) at 0 h, 12 h, 24 h, and 36 h. The gap was analyzed using ImageJ WH-NJ macro software (NIH, SUA) (Al-Zharani et al., 2020; Luanpitpong et al. 2010).

### 2.5 Mitochondrial Membrane Potential MMP (Δψm)

The MMP assay was performed using JC-1 dye (Abcam CAS), as described by Maqsood et al., (2020). Cells were grown in a medium and incubated as above. After 24 hrs, cells were exposed to 5-FU, each dose of lycopene and the mixture as mentioned earlier. JC-1 was used to stain the cells for 20 min. For monomer JC-1, the absorbance was measured at excitation: 475, emission: 530 nm while the aggregate JC-1 was read at undamaged mitochondria at excitation: 535, emission: 590 nm. Fluorescent images were analyzed under the confocal microscope (spinning disk, Zeiss, Germany). Cells showed up green fluorescent indicates monomer JC-1, in contrast, red fluorescent indicates aggregate JC-1.

### 2.6 TUNEL assay

Caco-2 cells were cultured on a slid cover in 6 wells plate and incubated at 37 °C for 24 hrs. After that, the cells were treated with drugs as above. This was followed by fixing with 1 % of paraformaldehyde and then washing with PBS. Cells were incubated in 70 % alcohol for 15 min, and then replaced with 51 µl of DNA labeling solution for 1hr at 37 °C, then 10 µl of Propidium Iodide/RNase staining buffer was added and incubated again for 30 min. Cells were washed with 1 ml of rinse buffer solution once followed by PBS. Cells were visualized under a confocal microscope (Spinning disk, Zeiss, Germany) (Almarzoug et al., 2020).

### 2.7 Annexin V-FTIC apoptosis staining and detection

Cells were cultured in a 6-well plate followed by incubation under standard conditions. Cells were exposed to 5-FU and lycopene as before. The medium was collected in a tube, and the cells were collected and centrifugated at 1500 rpm for 12 min at 20 °C. Cells were re-suspended with 500 µl of binding buffer with 5 µl of each of Annexin V-FITC and Propidium Iodide PI. Cells were protected from light for 15 min and then quantified according to Al-Salem et al. (2019).

### 2.8 Gene expression

RNA from treated cells was isolated using RNeasy Mini Kit (Cat No. /ID: 74104). Purified RNA samples were used for synthesizing the cDNA using cDNA kit (Thermofisher USA. Cat No.00881457). The efficiency of amplified PCR products was confirmed via 2 % of agarose gel. To determine the presence of the target sequence of apoptotic-related genes, RT-PCR was performed for *Bcl2, Bax, P53, Cas-3, 8*, and *9* after 24 h exposure to drugs. Sequences of primers used in RT-PCR were tabled in Table 1. The accumulation of amplification product is visualized with a fluorescence detector (Go Taq qPCR Master Mix, SYBR® green). The fold expression of the genes was calculated using the 2^- ΔΔCT method [where ΔCT = Ct (target) – Ct (β- actin) and ΔΔCt = ΔCt (treated) – ΔCt (Untreated)] (Peinnequin et al.,2004).

**Table 1:** Sequences of primers used in RT-PCR.

Primers	Forward	Reverse
*β-actin*	5′ TCACCCACACTGTGCCCATCTACGA 3′	5′ AGCGGAACCGCTCATTGCCAATGG 3′
*BCL2*	5′ AGGAAGTGAACATTTCGGTGAC 3′	5′ GCTCAGTTCCAGGACCAGGC 3′
*BAX*	5′ TGCTTCAGGGTTTCATCCAG 3′	5′ GGCGGCAATCATCCTCTG 3′
*Caspase 3*	5′ ACATGGCGTGTCATAAAATACC 3′	5′ CACAAAGCGACTGGATGAAC 3′
*Caspase 8*	5′ CTGGTCTGAAGGCTGGTTGT 3′	5′ CAGGCTCAGGAACTTGAGGG 3′
*Caspase 9*	5′ CCAGAGATTCGCAAACCAGAGG 3′	5′ GAGCACCGACATCACCAAATCC3′
*P53*	5′ CCCAGCCAAAGAAGAAACCA 3′	5′ TTCCAAGGCCTCATTCAGCT 3′

### 2.9 Statistical analysis

Obtained data were analyzed by one way of variance (ANOVA). However, the significant *P* values were had determined as ^#^*p* < 0.05, * *p* < 0.05, ** *p* < 0.01, *** *p* < 0.001.

## 3 Results

### 3.1 Cell viability

**Fig. 1:**
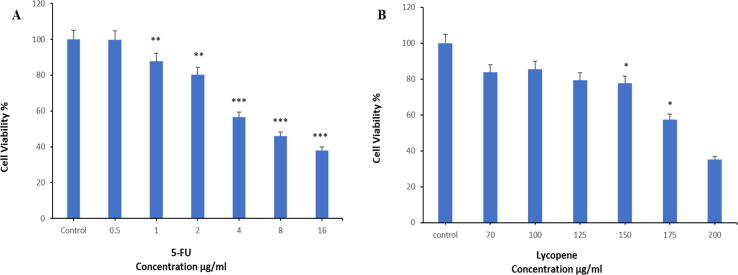
Cell viability of Caco2 cells after treatment with different concentrations of (**A**). 5-FU and (**B**). lycopene for 24 hrs as evaluated by MTT assay. Each value represents the percentage of cells viability. Each value represents the mean SE ± (n = 3), (*p < 0.05, **p < 0.01, ***p < 0.001) compared with control.

Growth inhibition and cytotoxic effect of tested compounds and their mixtures were examined. Caco2 cells were treated with 5-FU and with different concentrations of lycopene for 24 hrs. Fig. 1 A and B revealed that both 5-FU and lycopene resulted in a dose-dependent reduction in cell viability. A significant reduction in cell viability was observed for 5-FU at concentrations ≤1 µg/ml and 150 µg/ml for lycopene. IC_50_ of 5-FU was 6.1 µg/ml, and lycopene IC_50_ was 183.7 µg/ml. Based on this, a lycopene concentration of between 60 and 120 µg/ml that is not cytotoxic to the cell line was used for subsequent combinational dosing.

### 3.2 Lycopene mitigates 5-FU-induced cytotoxicity

**Fig. 2:**
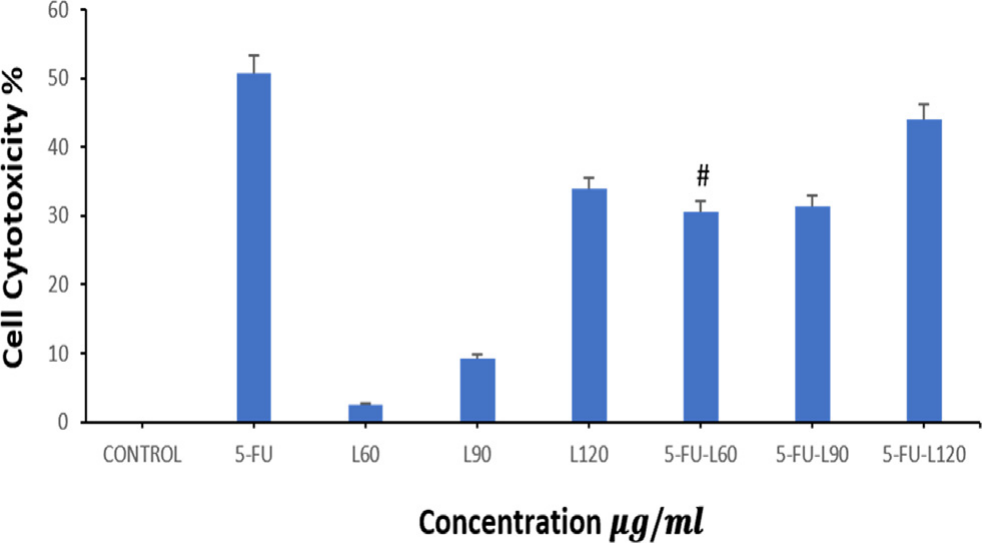
Cytotoxicity of 5-FU and lycopene on Caco2 cells after 24 hrs as evaluated by NRU assay. Each value represents the mean SE ± (n = 3).

To confirm cell survival, an NRU test was used. The dye determines the viable cells that have intact membranes. The movement of the lysosome enzymes to the outside of the cell due to membrane damage is monitored by quantifying and evaluating the dye. Caco2 cell line was exposed to 3 µg/ml of 5-FU, (60, 90, 120 µg/ml of lycopene), (mix of 5-FU with each of 60, 90, and 120 µg/ml of lycopene) for 24 h. As illustrated in (Fig. 2), 5-FU promoted cytotoxicity compared to lycopene doses alone. However, co-exposure of the cells to a mixture of lycopene to 5-FU ameliorates the cytotoxic effect of 5-FU for all mixture doses trialed. Interestingly, the mix of 5-FU with 60 µg/ml of lycopene significantly reduced the cytotoxic effect of 5-FU.

### 3.3 Caco2 cells migration

**Fig. 3:**
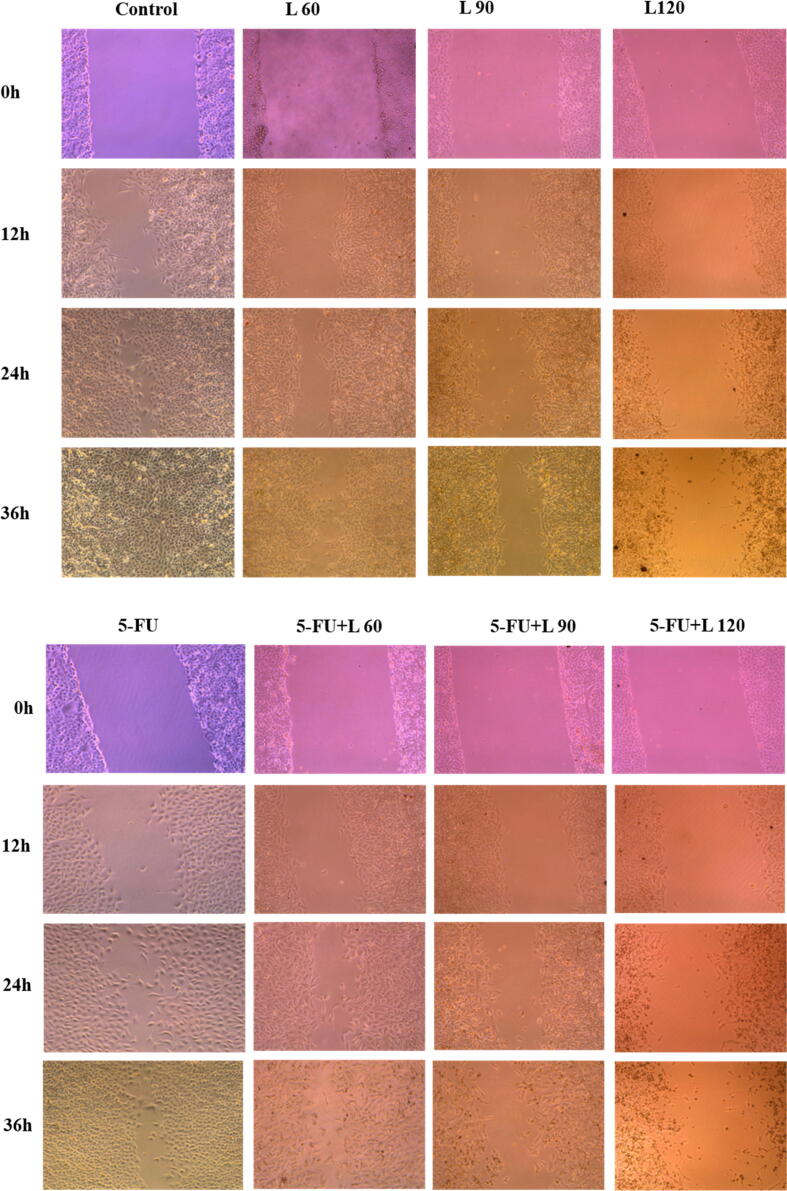

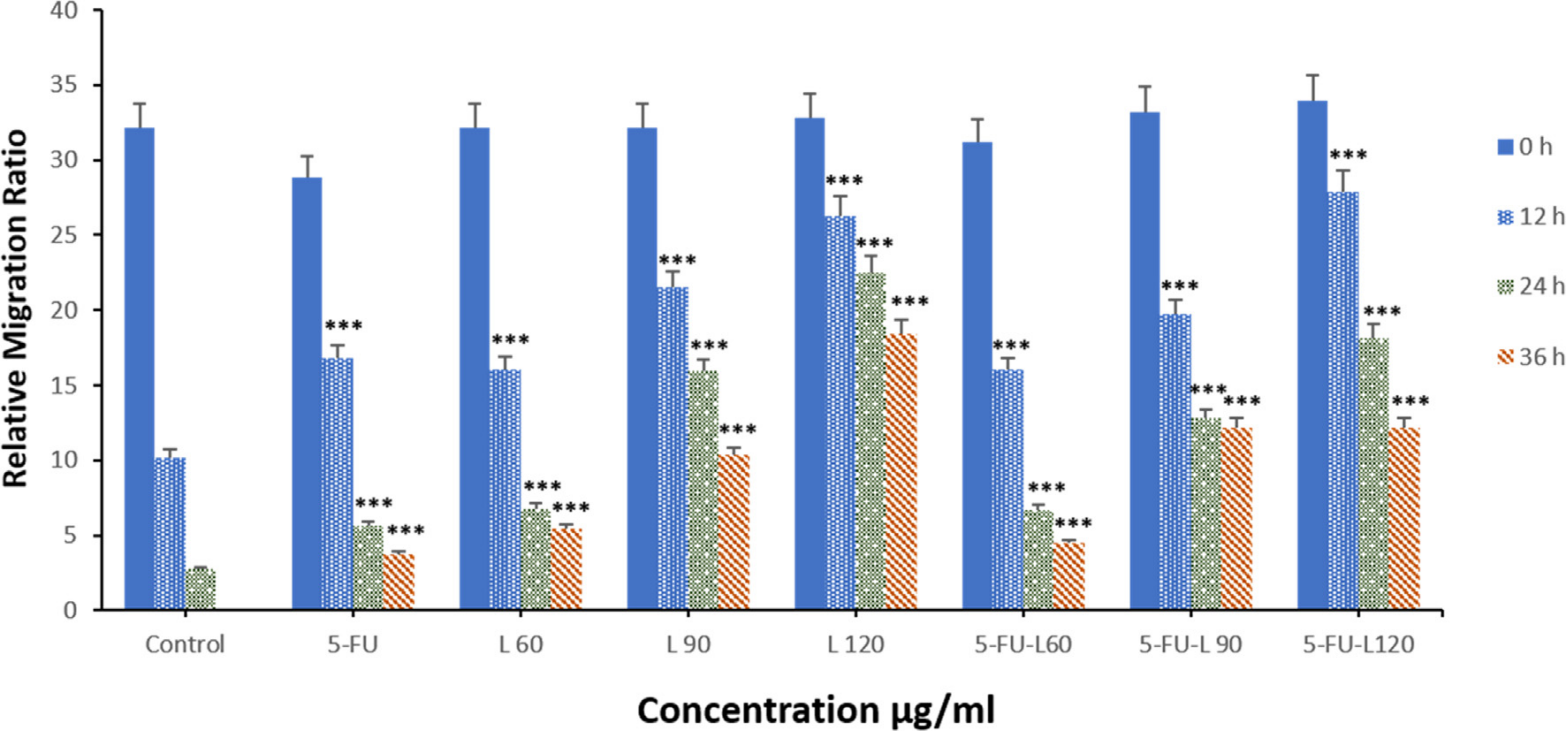
Caco2 cells were treated with 5-FU, L60, L90, L120, 5-FU + L 60, 5-FU + L 90, and 5-FU + L 120 for 24 hrs. (**A1 and 2**). Images were captured at 0 and 12, 24, and 36 hrs. Magnification (20X). (**B**). Caco2 relative migration rate in wound-healing assays. *** *p* < 0.001. vs control.

Cell migration was measured by wound healing assay. Caco2 cells were exposed to a dose of 5-FU and three doses of lycopene either alone or together with 5-FU for 24 hrs. As shown in Fig. 3 A1, A2, and B, there was a significant decrease in the rate of cell growth towards the generated gap. The reduced cell growth is dose- and time-dependent and was observed for all the drugs in comparison with the control cells. Contrastingly, treatment with 5-FU appeared to have very little effect on cell migration. This was, however, different in cells treated with a mixture of lycopene and 5-FU, where there was inhibition of migration in a dose-dependent manner.

### 3.4 Membrane potential (Δψm)

**Fig. 4:**
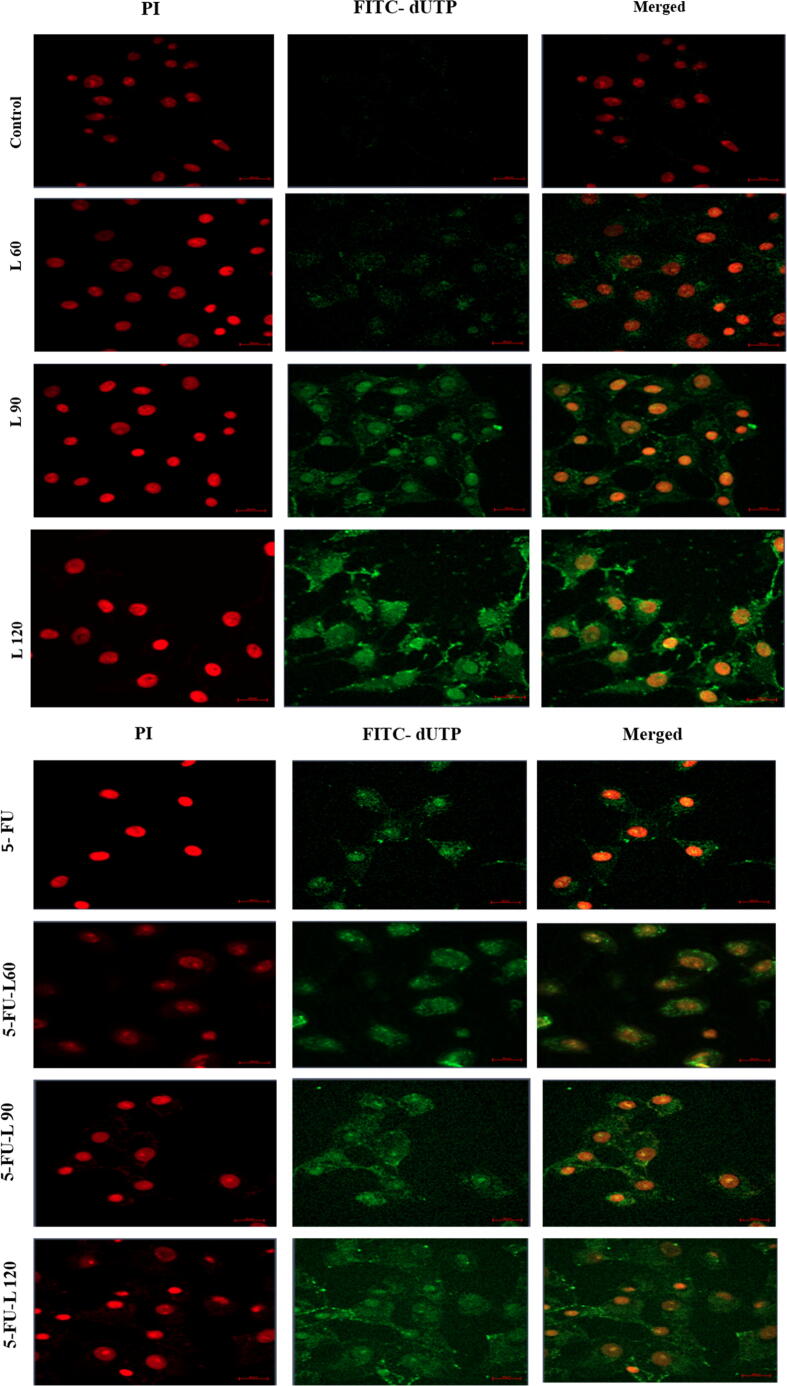

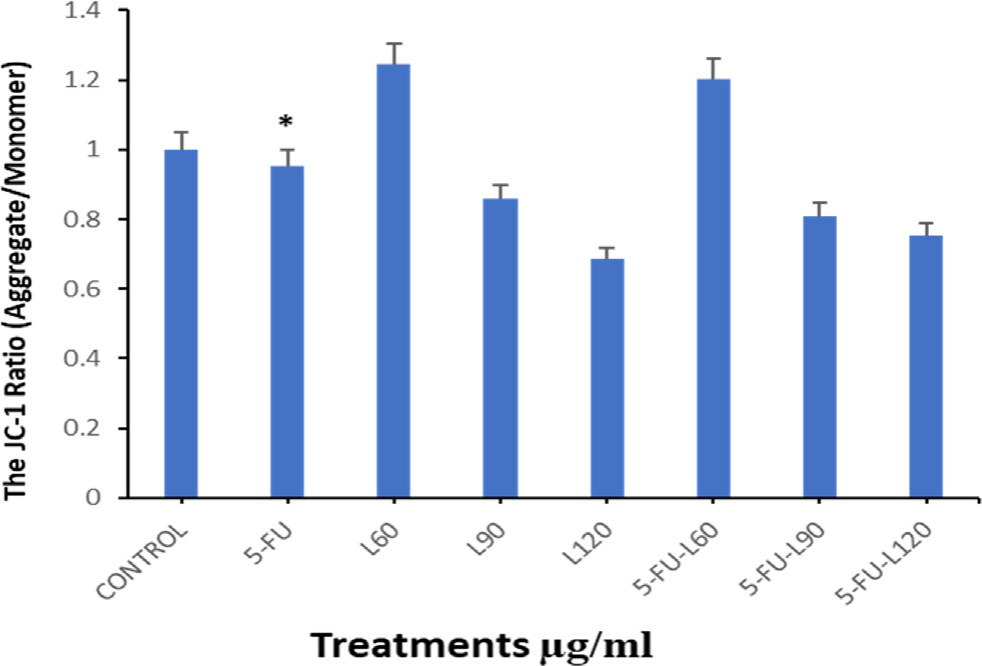
Shows MMP test of Caco2 cells treated with each compound for 24 hrs as evaluated by JC-1. (**A1**). cells treated with lycopene doses. (**A2**). cells treated with 5-FU alone and in combination with each dose of lycopene. Magnification (63X) and scale bar (20 µm). (**B**). The ratio of aggregate/monomer fluorescence intensity. Data are presented as the mean ± SD of three different experiments. *p < 0.05.

Monitoring mitochondria membrane potential is used to analyze mitochondrial membrane potential changes and apoptosis using two fluorescent dyes that stain healthy mitochondrion (high MMP) with red fluoresces and damaged mitochondria (low MMP) with green fluoresces. As illustrated in (Fig. 4A1, A2, and B) the reduction in the ratio of JC-1 aggregate/monomer for cells treated with L60,90,120 and M60,90,120 respectively but 5-FU had a significant decrease after 24 h incubation. However, treatment with L60,90,120 and M60,90,120 showed a decrease in MMP which indicates the cells may be undergoing apoptosis. Interestingly, 5-FU does not seem to influence the MMP. Lycopene alone, however, induced a similar reduction in the MMP when compared with the mixture doses of equivalent lycopene concentrations showing that lycopene may be the inducer of reduced MMP.

### 3.5 Lycopene enhances 5-FU-induced apoptosis

**Fig. 5:**
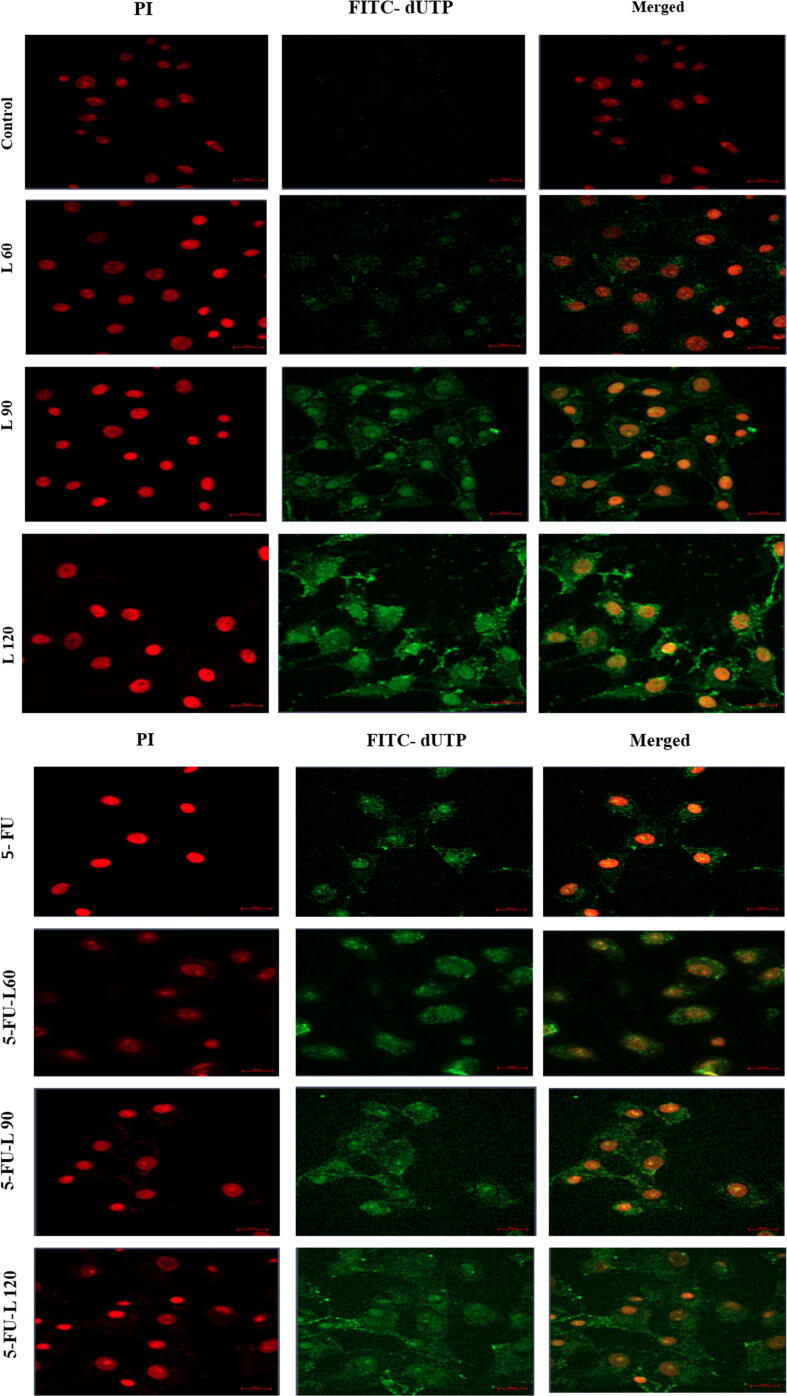

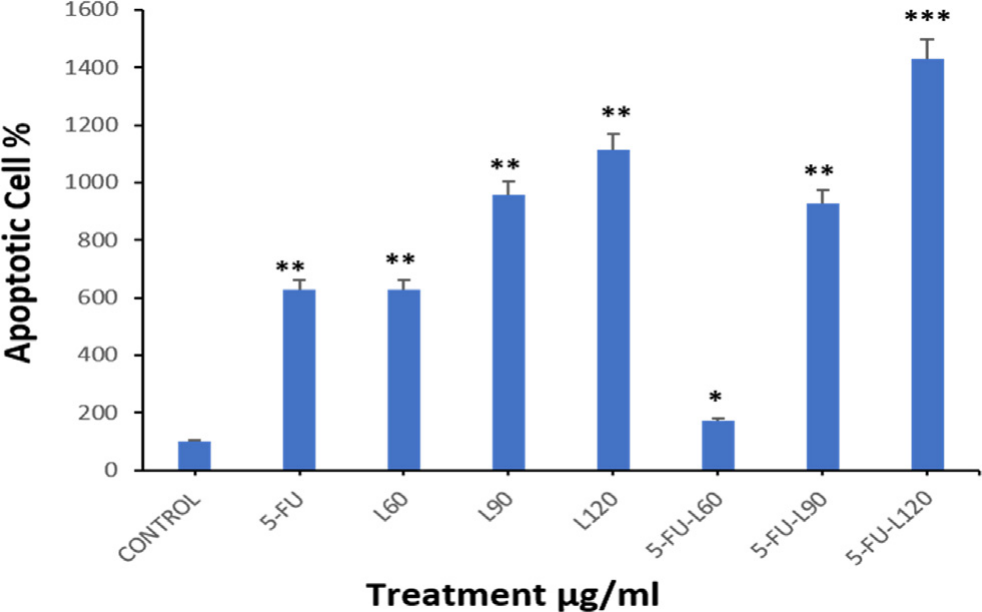
TUNEL assay results showed the apoptotic Caco2 cells after treatment with, (**5A1**). Three doses of lycopene. (**5A2**). 5-FU alone and in combination of three doses of lycopene. Magnification (63X) and scale bar (20 µm). (**5B**). The percentage of apoptotic cells. Each value represents the mean SE ± (n = 3), (*p < 0.05, **p < 0.01, ***p < 0.001).

To confirm apoptotic activation in the cells as shown in the findings from the evaluated MMP, the TUNEL assay was employed to evaluate degraded DNA in the exposed cells. As shown in (Fig. 5A1, A2, and B), a significant increase in the proportion of apoptotic cells was found after 24 hrs incubation with 5-FU, lycopene 60, 90,120, and a mix of 5-FU + L90. Interestingly, co-exposure to both lycopene and 5-FU resulted in higher DNA damage when compared with cells exposed to either 5-FU or lycopene alone.

### 3.6 Combinational treatment modulates apoptotic genes expression

**Fig. 6:**
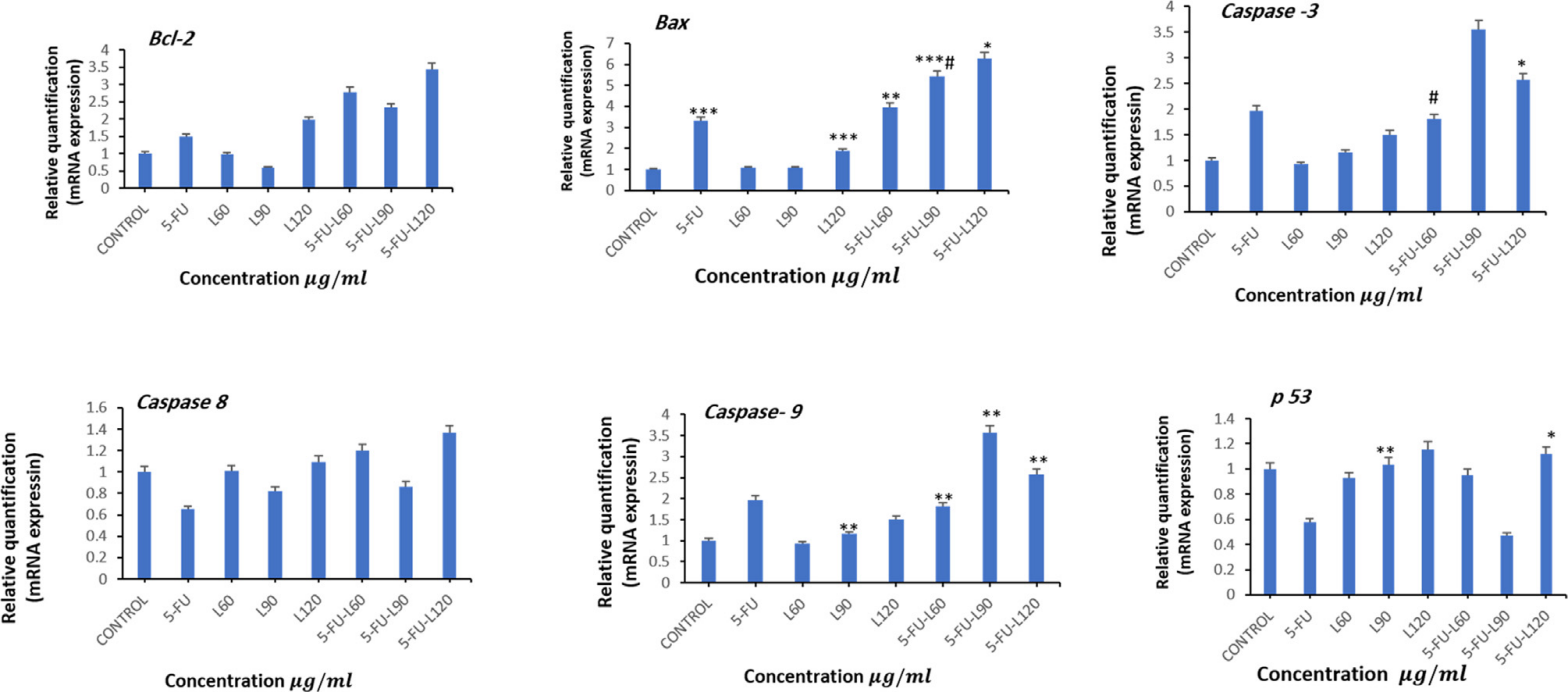
Shows fold change in the expression of apoptosis-related genes in treated Caco2 cells analyzed by real-time PCR (qPCR). Caco2 cells were exposed to different doses of lycopene, 5-FU, and mixture of 5-FU and Lycopene for 24 hrs. the measured genes were; *Bcl-2, Bax, Cas-3, Cas-8, Cas-9*, and *p53* respectively. The results are presented as the mean ± SD of three different experiments. Data represents the mean ± SE. n = 3, (*p < 0.05, **p < 0.01, ***p < 0.001 compared with control, #*p* < 0.05 compared with 5-FU.

Bax promotes apoptotic pathways to enhance apoptosis. Current results showed that *Bax* gene was significantly upregulated after exposure to 5-FU, 120 lycopene, mix 90, and mix 120 for 24 hrs. In contrast, lycopene 60 or 90 didn’t influence *Bax* gene expression. Furthermore, mix 90 significantly upregulated *Bax* expression compared with 5-FU (Fig. 6). Unlike Bax, Bcl-2 is an anti-apoptotic protein that works by inhibiting pro-apoptotic proteins. This study found *Bcl-2* was significantly upregulated after exposure to 120 lycopene, mix 60, Mix 90, and 5-FU. Interestingly, there was a downregulation in *Bcl-2* gene after exposure to mix 90 which increases the *Bax/Bcl-2*. The action of Bax and Bcl2 is to eventually activate Caspase activation where the cells should undergo apoptosis. On the contrary, exposure to lycopene 90, Mix 60, Mix 90, and Mix 120 led to significant upregulation in Cas-9. Meanwhile, Cas-8 is involved in the extrinsic apoptotic pathway where activation of Cas-3 is a result of activation of Cas-8. The results from our study showed an upregulation in Cas-8 after incubation with Mix 120 only. Cas-3 is an executioner caspase with a more direct role in apoptotic events. During apoptosis activation of Cas-8 and 9, followed immediately with activation of Cas-3. Our data showed upregulation in Cas-3 after exposure to lycopene 60, 90, and 120 for 24 hrs. However, a significant downregulation in Cas-3 was measured for Mix 120. With the Bax and Bcl-2 gene expression, mix 90 showed the highest Bax/Bcl-2 ratio in this treatment group, and upregulation of Cas-3 and Cas-9 were observed. Furthermore, mix 60 significantly downregulated *Cas-3* expression compared with 5-FU. *p53* as a tumor suppressor gene triggers apoptosis when DNA repair mechanisms fail by inhibiting the cell cycle progression. In this study, *p53* was found to be significantly upregulated after exposure to lycopene 90 and Mix 120 when compared to the control untreated cells.

### 3.7 Annexin V-FTIC apoptosis staining and detection

Annexin V-FTIC and PI are used to detect the percentage of apoptotic and necrotic cells. Flow cytometry analysis illustrated that Caco2 cell exposure to 5-FU significantly increased apoptotic cells. Furthermore, lycopene 120, Mix 60, Mix 90, and Mix 120 similarly increased apoptosis in the cells compared to untreated cells. In contrast, the percentage of necrotic cells was significantly higher after exposure to Mix120, and lycopene 120. Thus, the mixture induced more apoptosis compared to 5-FU (Fig. 7A, Fig. 7B).

**Fig. 7A:**
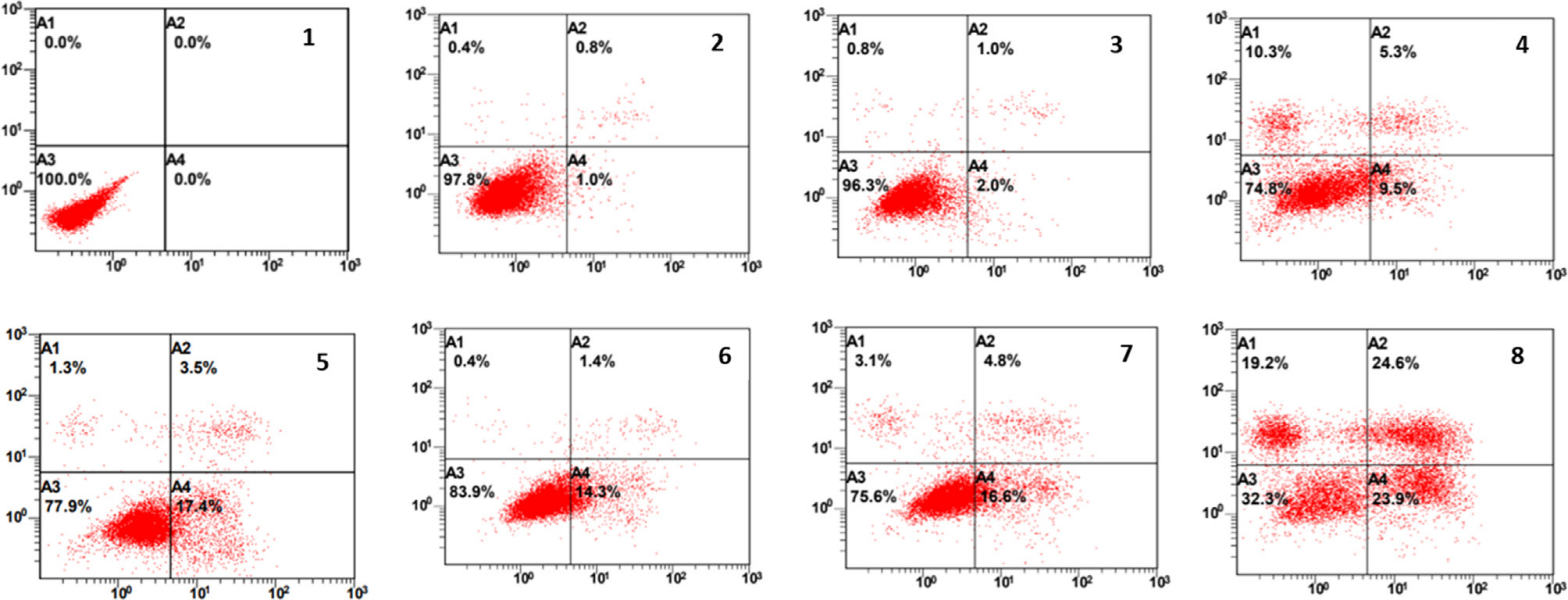
Shows flow cytometric analysis of Caco2 cells treated with each compound for 24 hrs. **1.** Control, **2.** L60, **3.** L90, **4**. L120, **5.** 5-FU, **6.** 5-FU + L60, **7.** 5-FU + L90, **8.** 5-FU + L120.

**Fig. 7B:**
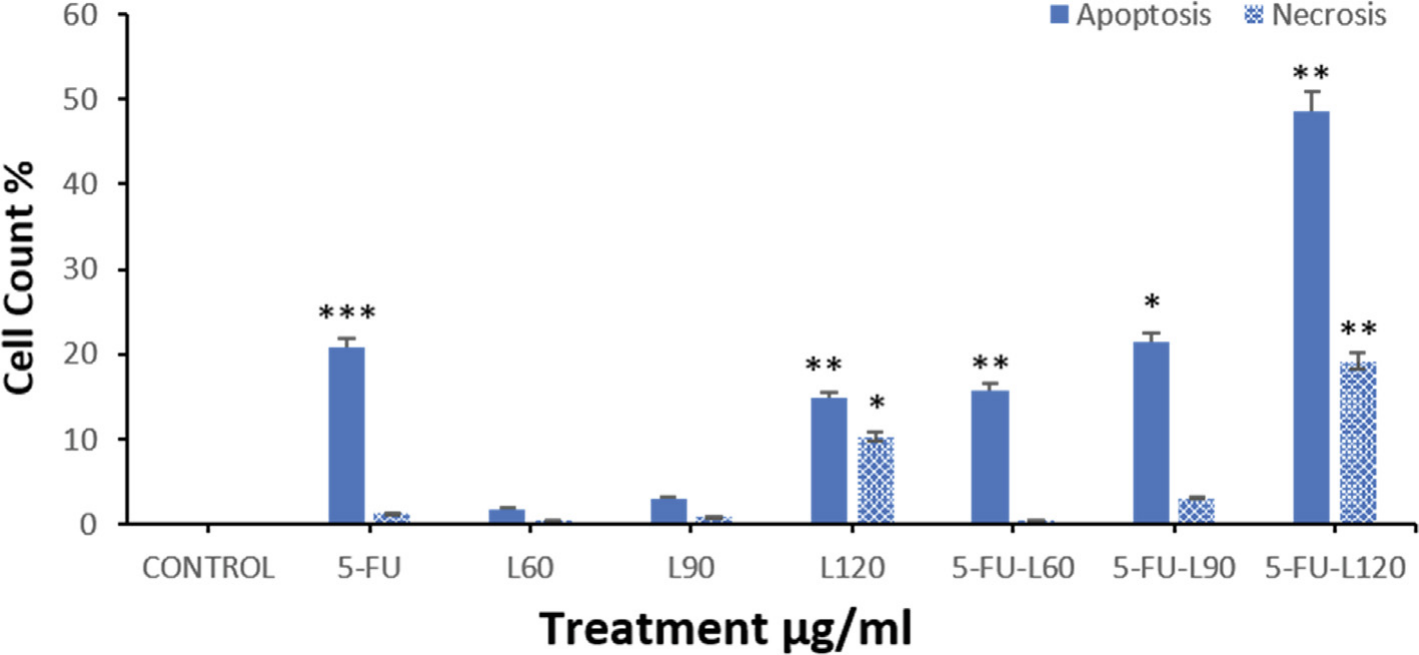
Shows flow cytometric analysis of Caco2 cells treated with each compound for 24 hrs. Data are presented as the mean ± SD of three different experiments. *p < 0.05, ** p < 0.01, *** p < 0.001 vs control.

## 4 Discussion

The most effective treatment for patients with late-stage of CC the surgical removal of the tumor tissue followed by adjuvant radiotherapy or chemotherapy to treat any leftover cancer cells. Furthermore, patients with early-stage cancer may also need adjuvant chemotherapy to enhance the primary chemotherapy. The setback to conventional chemotherapeutics used in CC, especially 5-FU, which is the first line of treatment of CC, is the development of resistance by cancer cells (Blondy et al., 2020). Thus, it is important to develop adjuvant chemotherapeutics that can enhance the cancer-killing effect of 5-FU as well as suppress the side effects that may be associated. Few studies investigated the effect of the combination of lycopene, sulforaphane, quercetin, and curcumin ingredients on daily diet (Qi et al., 2021). Another study was conducted on different colon cancer cell lines such as; colon epithelial (CCD841 CoTr and HT-29, LS174T) (Qi et al., 2021), but not Caco2 cell line that we have investigated. So, our novel finding is applying different concentrations of lycopene combined with 5-FU.

Current research work evaluated the anticancer properties of lycopene as an adjuvant therapy with 5-FU in the treatment of CC using Caco2 cancer cell line. Our findings showed that 5-FU induced cytotoxic which is consistent with that of several other studies (Ikehata et al., 2014, Udofot et al., 2015, Eynali et al., 2017). The cytotoxicity induced by 5-FU is often accompanied by inflammation that is damaging to tissues. 5-FU induces gastrointestinal inflammation-causing intestinal damage at both local and distal sites in an *in vivo* rat model (Soares et al., 2008, Soares et al., 2013). *In vitro*, 5-FU induces a significant increase in levels of hepatic triglyceride, which is accompanied by dysfunction of mitochondria (MT) and elevates the level of fatty acyl-CoA oxidase 1 (ACOX1), which causes oxidative stress (Sommer et al., 2017). Another study demonstrated that 5-FU and Oxaliplatin have different effects on HCT116 and SW620 cell lines and their chemoresistant sublines. Treatments with 5-FU and Oxaliplatin significantly modulate protein levels of core-autophagy proteins ATG7 and ATG12 (Zitkute et al., 222)). A combination of STH with 5-FU was applied to treat colon cancer cells and could lead to fewer effective doses and consequently lower side effects. The molecular mechanisms were associated with the suppression of cell proliferative markers and apoptosis induction (Afrin et al., 2021).

Lycopene is an antioxidant and also known to have an anti-inflammatory property and has been reported to induce an anti-cancer effect through the resolution of inflammation caused by an azoxymethane-induced pre-neoplastic lesion, aberrant crypt foci, and inflammatory biomarkers, COX-2 and iNOS (Sengupta et al., 2006). Lycopene also promotes the antioxidant enzyme activities of catalase, glutathione peroxidase, and SOD (Luo and Wu, 2011). The activity of lycopene against inflammation may mediate the toxic effects of 5-FU. While 5-FU induced a significant level of apoptosis, we found higher induction of apoptosis and necrosis in lycopene-only exposed cells compared to 5-FU. This was further increased in combination treatment of 5-FU and lycopene for all mixtures indicating the increase in both cell death forms was due to lycopene exposure. This activity of lycopene on apoptosis induction is in agreement with studies that showed lycopene to induced necrosis and apoptosis in CC cell lines (Lin et al., 2011, Huang et al., 2015). This enhancement might be because of the antiinflammatory effect of lycopene.

Anticancer effects of lycopene have been previously attributed to its anti-inflammation effects as it helps annul cancer-promoting inflammation. Lycopene dose-dependently suppressed IL-1β, IL-6, TNF-α, iNOS, and COX-2 gene expression in a human CC cell line (Cha et al., 2017). The intrinsic apoptotic pathway involves the release of cytochrome *C* from the MT leading to the eventual activation of executioner caspases 3 and 7 (Yusuf et al., 2018). We first investigated MMP to investigate the pathway of apoptosis that is activated. We found lower MMP in cells exposed to a combination of 5-FU and lycopene mixture containing lycopene at 90 and 120 µg/ml. This indicates an unstable mitochondrial membrane, which is induced by pore formation of Bax on the mitochondrial membrane. Bax is regulated by Bcl-2 binding which prevents it from forming pores on the mitochondrial membrane. As such, induction of apoptosis is determined by the ratio of Bax to Bcl-2 in the cell (Yusuf and Casey, 2020, Al-Johani et al., 2022). Higher Bax/Bcl-2 ratio promotes apoptosis and this was reported in this research work, especially for the Mix 90 but not with 5-FU. MT outer membrane permeabilization (MOMP) is caused due to low MMP and this is a consequence of pore formation of Bax on the mitochondrial membrane allowing the release of cytochrome *C*. DNA damage enhancing *p53* to activate pro-apoptotic genes such as *Bax* that induce releasing of cytochrome *C* out of MT to initiate intrinsic pathway (Etti et al., 2017). Taken together, the released cytochrome *C* is used in the building up of the apoptosome complex and subsequent activation of Cas-9 which then activates Cas-3. Interestingly, cells treated with Mix 90 exhibited the highest expression of caspase 3 and 9. As such lycopene seem to have triggered Bax expression in the mixture subsequently resulting in activation of apoptotic events. Metastasis causes about 90 % of cancer-related death and it is driven by the ability of cancer cells to migrate from the primary growth site to distal secondary sites (Seyfried and Huysentruyt, 2013).

Present data revealed that 5-FU treatment did not induce a significant effect on cell migration. However, we found that exposure to lycopene alone and its mixture dose-dependently inhibited the cell migration. This is likely due to the induced apoptosis which inhibits cell proliferation and eventual migration. Taken altogether, it seems that lycopene sensitizes the cells to treatment with 5-FU since there was slow mRNA transcription of apoptotic genes and observed apoptosis, as well as unaffected cell proliferation and migration by 5-FU alone, compared to the mixture containing lycopene.

## 5 Conclusion and future work

We have shown here that lycopene sensitizes Caco2 cell lines to 5-FU by inducing the expression of apoptotic genes. This in addition, to the suppression of cytotoxicity and cell migration by lycopene indicates lycopene is a promising candidate for adjuvant therapy involving 5-FU in CC. While 5-FU still induced a cytotoxic effect, there was a less pronounced effect on apoptosis and cell migration which indicates the potential of the cancer cells to metastasize. This highlights the need for adjunct therapy that can be used to sensitize CC cells to conventional drugs such as 5-FU to broaden its application and benefits to more patients. Future studies should investigate inflammatory markers and the mechanisms of lycopene in animal models.

## Authors' contributions

Norah M. Alhoshani and Norah S. Al-Johani performed the cytotoxic and MMP assays. Norah M. Alhoshani and Saad Alkahtani assessed the DNA damage. Norah M. Alhoshani measured the cell migration. Saud Alarifi and Nora Alkeraishan analyzed flow cytometry data. Norah S. Al-Johani and Nora Alkeraishan performed RNA isolation and cDNA synthesis. Norah M. Alhoshani and Saud Alarifi measured the gene expression.

## Declaration of Competing Interest

The authors declare that they have no known competing financial interests or personal relationships that could have appeared to influence the work reported in this paper.
